# Methionine tumor starvation by erythrocyte‐encapsulated methionine gamma‐lyase activity controlled with per os vitamin B6

**DOI:** 10.1002/cam4.1086

**Published:** 2017-05-23

**Authors:** Fabien Gay, Karine Aguera, Karine Sénéchal, Angie Tainturier, Willy Berlier, Delphine Maucort‐Boulch, Jérôme Honnorat, Françoise Horand, Yann Godfrin, Vanessa Bourgeaux

**Affiliations:** ^1^ ERYTECH PharmaLyonFrance; ^2^ VoxcanMarcy l'EtoileFrance; ^3^ Service de BiostatistiqueHospices Civils de LyonLyonFrance; ^4^ Université Claude Bernard Lyon 1VilleurbanneFrance; ^5^ CNRS UMR 5558Laboratoire de Biométrie et Biologie EvolutiveEquipe Biostatistique‐SantéVilleurbanneFrance; ^6^ Service de Neuro‐oncologieHôpital neurologiqueHospices Civils de LyonLyonFrance; ^7^ Institut NeuroMyoGene INSERM U1217/CNRS UMR 5310LyonFrance

**Keywords:** Erymet, methionine‐dependent cancers, methionine gamma‐lyase, pyridoxal 5′‐phosphate, red blood cells

## Abstract

Erymet is a new therapy resulting from the encapsulation of a methionine gamma‐lyase (MGL; EC number 4.4.1.11) in red blood cells (RBC). The aim of this study was to evaluate erymet potential efficacy in methionine (Met)‐dependent cancers. We produced a highly purified MGL using a cGMP process, determined the pharmacokinetics/pharmacodynamics (PK/PD) properties of erymet in mice, and assessed its efficacy on tumor growth prevention. Cytotoxicity of purified MGL was tested in six cancer cell lines. CD1 mice were injected with single erymet product supplemented or not with vitamin B6 vitamer pyridoxine (PN; a precursor of PLP cofactor). NMRI *nude* mice were xenografted in the flank with U‐87 MG‐luc2 glioblastoma cells for tumor growth study following five intravenous (IV) injections of erymet with daily PN oral administration. Endpoints included efficacy and event‐free survival (EFS). Finally, a repeated dose toxicity study of erymet combined with PN cofactor was conducted in CD1 mice. Recombinant MGL was cytotoxic on 4/6 cell lines tested. MGL half‐life was increased from <24 h to 9–12 days when encapsulated in RBC. Conversion of PN into PLP by RBC was demonstrated. Combined erymet + PN treatment led to a sustained Met depletion in plasma for several days with a 85% reduction of tumor volume after 45 days following cells implantation, and a significant EFS prolongation for treated mice. Repeated injections in mice exhibited a very good tolerability with only minor impact on clinical state (piloerection, lean aspect) and a slight decrease in hemoglobin and triglyceride concentrations. This study demonstrated that encapsulation of methioninase inside erythrocyte greatly enhanced pharmacokinetics properties of the enzyme and is efficacy against tumor growth. The perspective on these results is the clinical evaluation of the erymet product in patients with Met starvation‐sensitive tumors.

## Introduction

Cancer‐associated reprogramming of cellular metabolism was recently reconsidered as a core hallmark of cancer 1. This metabolism deregulation forces cancer cells to have significant needs in terms of energy and biosynthetic intermediates in order to sustain their higher proliferation rate, leading to a strong dependency on several nutrients present in the tumor microenvironment. This required supply of exogenous nutrients (i.e., auxotrophy), not observed in normal cells, has long been considered a promising field for the development of cancer therapies, and in recent years, there has been renewed interest in altered cancer metabolism pathways 2, 3, 4, including the emergence of amino acid metabolism as a promising target 5.

l‐Methionine (Met) is a dietary essential, sulfur‐containing proteinogenic amino acid involved in several cellular metabolism pathways 6.

Met auxotrophy was observed in several xenograft models 7 and in addition, dietary Met was reported to affect intestinal carcinogenesis in vivo: development of adenomas was enhanced by 41% in Apc^Min/+^ mice fed a normal diet supplemented with 0.7% additional Met for 4 weeks 8.

Met auxotrophy was also reported in 25% of fresh and cultured patient tumors from different organs 9. Interestingly, imaging of Met incorporation by tumor cells using ^11^C‐MET positron emission tomography is also used in the clinical management of glioblastoma, highlighting the eagerness of aggressive brain tumor cells for Met 10.

As Met restriction appeared to be appropriately selective of tumor cells, two different therapeutic approaches were considered for Met depletion at the systemic level using nutritional starvation or Met‐catabolizing enzymes (see 11, 12, 13 for complete reviews).

A Met‐free diet was abundantly tested in various preclinical tumor‐bearing models, mainly via parenteral nutrition, and confirmed its efficacy to induce tumor regression, alone or in combination with standard chemotherapeutic drugs 14, 15.

Met‐depleted parenteral nutrition was also tested in a prospective clinical trial in advanced gastrointestinal tract cancer and after 7 days of a Met‐free diet administered concomitantly with 5‐FU, resected tumors harbored histological evidence of efficacy, as compared to tumors from control patients treated with 5‐FU alone 16. Several phase I clinical trials of dietary Met restriction, alone or in association with chemotherapeutic drugs as cystemustine or FOLFOX regimen, were carried out in patients with aggressive or refractory solid tumors and demonstrated feasibility and good safety of Met‐free diet on short period (1–3 days every 2 weeks) 17, 18. In all these clinical studies, the initial Met plasma level decreased by 55% to 58%. Although Met starvation led to efficient systemic depletion of the amino acid and despite good compliance, patients encountered difficulties in maintaining sufficient energy needs, even with regular dietitian follow‐up, and were prone to weight loss (up to 0.5% body mass index weight per week), early satiety, and lack of appetite, showing the difficulty of administering these regimens over the long term 17, 18, 19.

A second promising strategy is based on the use of the Met‐cleaving enzyme as methionine gamma‐lyase (MGL; EC number 4.4.1.11). MGL is a pyridoxal 5′‐phosphate (PLP)‐dependent enzyme involved in the metabolism of Met. This enzyme is ubiquitous in all organisms, except in mammals. MGL specifically degrades Met to alpha‐ketobutyrate, ammonia, and thiols. Due to its enzymological properties, MGL was isolated from several bacteria, fungi, and protozoa, but the most studied form was purified from *Pseudomonas putida*, which displays high affinity for Met and high catalytic efficiency 20.

Recombinant MGL tumor growth inhibitory efficacy has been verified in numerous cancer cell lines, and its cytotoxic effect was far more significant in these cell lines than in human nonmalignant cells 21, but its first major limitation is a short half‐life (~2 h) in the blood stream 22. The addition of PEG moieties extended enzyme half‐life in mice to 38 h, but frequent injections are still necessary for maintaining a depletion > 50% of physiological value 23, 24.

Even if pegylation has partially solved the problem of MGL half‐life, low bioavailability of enzyme cofactor PLP remains a major problem. MGL catalytic activity requires PLP, the biological active form of vitamin B6 as a cofactor. PLP is rapidly bound to plasma proteins, which limits its bioavailability. In addition, PLP's half‐life in the blood stream is only ∼15 min 25, 26. However, in vitro and in vivo studies have revealed that pyridoxine (PN), one vitamin B6 vitamer, can easily cross RBC membrane to be rapidly converted to PLP via an enzymatic reactions cascade 27. As semiautomated osmotic‐based encapsulation methods in RBC are now fully compatible with both industrial and clinical requirements 28, this particular characteristic of erythrocytes in providing biologically active PLP was used to develop an innovative therapy for the treatment of Met‐dependent cancers based on RBC‐encapsulated MGL.

The aims of the present study were (1) to produce highly purified MGL on an industrial scale and to verify its in vitro cytotoxic effects, (2) to evaluate the PK‐PD characteristics of MGL‐loaded RBC in healthy mice, (3) to assess the role of RBC in PLP biosynthesis from the exogenous uptake of PN, and (4) to investigate the antitumoral effect of MGL‐loaded RBCs in a subcutaneous xenografted mouse model bearing glioblastoma tumors.

## Materials and Methods

### Chemicals and reagents

Ammonia assay kit was from Roche Diagnostics (Meylan, France). Vitamin B6 enzymatic assay kit was obtained from Bühlmann Laboratories AG (Schönenbuch, Switzerland). Cell Counting Kit‐8 (CCK‐8), Met, and PLP were from Sigma (Saint‐Louis, MO). PN hydrochloride solution was from DB Pharma (La Varenne Saint Hilaire, France). Culture reagents were obtained from GE Healthcare Europe, GmbH, (Velizy‐Villacoublay, France), Invitrogen (Carlsbad, CA), and Sigma.

### MGL biomanufacturing

MGL coding sequence from *P. putida* was codon optimized for expression in *Escherichia coli*, synthesized, and cloned. The cGMP runs were conducted at a 350L fermentation scale. After two precultures (6 and 16 h) at 37°C in GY medium–glucose–kanamycin, and cooling at 28°C, protein expression was induced by 1 mmol/L IPTG. Cell sediment was harvested after 20 h and concentrated using a 500‐kDa hollow fiber. Cell pellet was recovered by centrifugation at 15,900*g* and suspended in lysis buffer (7 v/w). Lysis was performed at 10°C by high‐pressure homogenization at 1000, 600, and 600 bars. Lysate underwent clarification at 10°C by adding 0.2% PEI and centrifugation at 15,900*g*. The soluble fraction was sterilized by 0.2‐*μ*m filtration before precipitation with ammonium sulfate (60% saturation) at 6°C, over 20 h. Two crystallization steps (PEG‐6000 at 10% and ammonium sulfate at 10% saturation and PEG‐6000 at 12% and 0.2 mol/L NaCl at 30°C) were then carried out. After centrifugation (15,900*g*), pellet was resuspended in a solubilization buffer, filtered (0.45 *μ*m), and subject to two anion exchange chromatography (DEAE sepharose FF) steps. A polishing step (Q membrane chromatography) led to removal of contaminants. Purified MGL was concentrated and diafiltered in formulation buffer (50 mmol/L Na phosphate, pH 7.2; 20 *μ*mol/L PLP; 130 mmol/L NaCl) using a 10‐kDa cutoff TFF cassette, aliquoted, and stored at −80°C.

### Cell culture

AGS (ATCC CRL‐1739) and NCI‐N87 (CRL‐5822) human gastric adenocarcinoma, LN‐229 (CRL‐2611) and U‐87 MG (HTB‐14) human glioblastoma, and BxPC‐3 (CRL‐1687) and MIA PaCa‐2 (CRL‐1420) pancreatic carcinoma cell lines were grown according to ATCC recommended culture conditions. U‐87 MG‐luc2 cells (ref. 124577) were cultured following Caliper Life Sciences recommendations. Complete culture mediums contained 60–200 *μ*mol/L Met. All studies were performed with mycoplasma‐free cells in logarithmic phase of growth and blank wells and viability control were included. Cell density, CCK‐8, and MGL incubation times were optimized for each cell line.

### Cell proliferation assay

Cells at a fixed concentration were prepared in 100 *μ*L complete medium or Met‐deprived medium ± physiological dose of Met (40 *μ*mol/L) and dispensed in a 96‐well plate. All conditions were tested in duplicate. Plates were incubated for 24 h for cell seeding and cell viability measurements, and renewal of medium were carried out every 24 h for 10 days. For each time point, 10 *μ*L of CCK‐8 solution was added to each wells and plates were incubated for 1–4 h. Optical density (O.D.) was measured at 450 nm and percentage of growth was determined.

### Cytotoxicity assay for MGL

Cells cultured in complete medium (100 *μ*L) were dispensed in 96‐well plates at a fixed concentration. All conditions were tested in triplicate. Cells were incubated for 24 h. Then, 10 *μ*L of MGL, ranged from 0.001 to 14.6 U/mL (final concentrations), were added. After a 3 or 4 days incubation step, 10 *μ*L of CCK‐8 solution were added and plates were incubated for 1–4 h. O.D. was then read at 450 nm. The concentration that gives a 50% inhibition of cell viability (IC_50_) was determined using Gen5^™^ Data Analysis Software (BioTek, Winooski, Vermont, United States).

### Encapsulation process

MGL was loaded into mouse RBC by reversible hypotonic dialysis with Mini‐module M10 hollow fiber dialyzer (Gambro, Deerfield, IL). RBC were collected from mouse blood after centrifugation and plasma/buffy coat was removed and washed twice with 0.9% NaCl (v:v) solution. MGL was added to required concentration, leading to a suspension of 65% hematocrit (Ht). RBC were dialyzed against a hypotonic buffer of 40 mOsmol/kg and resealed by addition (10% v:v) of a hypertonic solution (detailed in 29) and then incubated for 30 min. RBC were then fluorescently tagged using CFDA‐SE labeling method, washed with 0.2% glucose 0.9% NaCl solution, and subjected to an incubation step at 37°C to eliminate more fragile RBC. After washing, the product was resuspended and adjusted to 50% Ht with SAG‐Mannitol 6% BSA or SAG‐Mannitol 20% plasma, stored at 2–8°C, and injected within 16 h. For batch release, erymet products were characterized for potency with total MGL content (0.55–1.05 mg/mL) and extracellular MGL (<9%) as well as for safety with the evaluation 24 h after manufacturing of extracellular hemoglobin level (<1.5 g/dL) and Ht yield (<95%).

### Animals

Male CD1 mice (Crl:CD1(ICR)) were from Charles River Laboratories. Male NMRI *nude* mice (Rj:NMRI‐*Foxn1* ^*nu*^ */Foxn1* ^*nu*^) were from JANVIER Labs. Animals (5 weeks old) were allowed to acclimatize for 7 days, identified, weighed, and randomly assigned to study groups. Animals were cared for in an accredited facility (Institut Claude Bourgelat, Marcy l'Etoile, France; approval number No. A 69 127 0505) in accordance to ethical guidelines 30. Animals had access to food and water ad libitum. A04 diet (SAFE) contained 2800 mg/kg of Met and 2.6 mg/kg of PN.

### Pharmacokinetics

Fluorescently labeled erymet products were injected intravenously into CD1 mice. At various time points, mice were sacrificed and blood was collected in lithium heparinate tubes kept at +4°C away from light. The proportion of fluorescent RBC in whole blood was determined by flow cytometry: 5 *μ*L of whole blood diluted in 1 mL of PBS, 0.5% BSA, and passed in triplicate (counting of 10,000 cells in FL‐1; FC500 cytometer, Beckman Coulter). Fluorescent RBC proportion was plotted as a function of time following erymet administration, and half‐life was determined by exponential regression.

### Pharmacodynamics

CD1 mice were injected with erymet product(s) ± PN administration or with free MGL enzyme and were sacrificed at different time points. Blood was collected in heparinized tubes kept at +4°C and rapidly centrifuged (10 min; 4°C) at 1000*g*. Plasma was then aliquoted and stored at −80°C until Met quantification.

### In vivo antitumor efficacy

Twenty NMRI *nude* mice were subcutaneously injected (day 0) with 3 × 10^6^ U‐87 MG‐luc2 cells in 100 *μ*L 1× DPBS and were randomly divided into two groups of 10 mice. Treated group received five IV injections of erymet (8 mL/kg, corresponding to 76 U/kg) on days 9, 16, 23, 30, and 37. Control group received SAG‐Mannitol–20% plasma vehicle. Nonimplanted mice (*n *= 12) were used to verify plasma Met depletion of erymet products, 24 h postinjections, and after the five treatments. From day 9 to day 45, mice received daily PN dose (100 *μ*L; 8.4 mmol/L) by oral route. Tumor growth was followed by caliper measurement and bioluminescence imaging. Tumor volume (TV; mm^3^) was determined by the standard formula TV = (L × W^2^)/2. For imaging, each mouse was injected i.p. with 300 *μ*L of luciferin. Animals (under isoflurane/oxygen anesthesia) were placed in an optical imaging system (IVIS 200 Series, Xenogen) and measurements were performed within an optimum time frame determined by kinetic acquisitions on three mice. Mice were weighed once a week and examined frequently for drug‐related side effects. Animals were euthanized if (1) TV > 2000 mm^3^, (2) loss of body weight > 20% to the maximal body weight, (3) appearance of tumor necrosis and/or metastases, or (4) severe deterioration of the general status.

### Dose–response antitumor effect in mice

Thirty‐six NMRI *nude* mice were subcutaneously injected (day 0) with 3 × 10^6^ U‐87 MG‐luc2 cells in 100 *μ*L 1× DPBS and were randomly divided into three groups of 10 mice. Treated groups received five IV injections of erymet (30 or 45 U/kg) on days 8, 15, 22, 29, and 36. Control group received SAG‐Mannitol–20% plasma vehicle. PN cofactor (8.4 mmol/L) was administered orally in combination with erymet or vehicle from day 8 to day 43. Tumor growth was followed by caliper measurement.

### In vivo evaluation of erymet toxicity

Evaluation of erymet toxicity was carried out at Voxcan (Marcy l'Etoile, France). Ninety‐six CD1 mice (males and females) were used to investigate induction of side effects. Treated groups (*n* = 48) received four IV injections of erymet (63 U/kg) on days 1, 8, 15, and 22, and daily administration of PN from day 2 to day 22. Control groups (*n* = 48) received SAG‐Mannitol–20% plasma vehicle as reference item. Mice were daily observed throughout the study and erymet toxicity was investigated by evaluating (1) mice clinical state using a scored clinical follow‐up, (2) plasma methionine concentration, and (3) hematological and serum clinical chemistry parameters analysis, at both day 23 (day following end of treatment period) and day 37 (15 days after treatment period). Animals were euthanized if (1) loss of body weight > 20% to the maximal body weight or/and (2) total clinical score > 4.

### Hematological profile and blood chemistry analyses

Before euthanasia, whole blood was sampled by retro‐orbital way in anesthetized mice (isoflurane 2–3%) and deposited in EDTA‐K2 or serum separator tubes. Hematological parameters included red blood cell count, hemoglobin (Hb), packed cell volume, mean corpuscular volume, mean corpuscular Hb, mean corpuscular Hb concentration, reticulocyte count, platelet count, total white blood cell count, and differential white blood cell count. Serum clinical chemistry parameters included analysis for sodium, potassium, chloride, calcium, glucose, urea, total cholesterol, triglycerides, total bilirubin, total protein, albumin, globulin, creatinine, alkaline phosphatase, aspartate aminotransferase, and alanine aminotransferase.

### Plasma Met quantification

Met level in murine plasma samples was quantified according to a RPLC‐ESI‐MS/MS validated method described by Piraud et al. 31. Basically, samples (75 *μ*L) were deproteinized with 300 *μ*L of methanol. After 2 min of vortex, and 5 min at room temperature, the samples were centrifuged (8 min at 17,000*g*). To 200 *μ*L of supernatant, 200 *μ*L of 1 mmol/L TDFHA containing a stable radioisotope of Met (methyl‐D3; Euriso‐top) as internal standard were added. Five *μ*L were injected on Api 3200 (AbSciex) tandem mass spectrometer.

### MGL activity assay

The assay is based on measurement of NH_3_ produced by MGL and assayed indirectly by enzymatic action of glutamate dehydrogenase (GLDH) according to a commercial kit (Roche). MGL standards were prepared in matrices (total RBC or supernatant) or in an aqueous solution. Alpha‐ketoglutarate, NADPH, and GLDH were added to samples or standards for endogenous NH_3_ removal. After 10 min, 75 *μ*L of Met (78.3 mmol/L) were introduced and reaction mixtures were incubated for 30 min. Degradation of NADPH into NADP+ was continuously tracked by measuring the O.D. at 340 nm. Percentage of MGL holoenzyme fraction was determined by dividing MGL activity measured without PLP supplementation by MGL activity measured with addition of PLP in the assay.

### PLP measurement

Quantitative determination of PLP concentration in plasma and whole blood was assessed by an enzymatic assay (Bühlmann). Briefly, tyrosine was converted to tyramine and CO_2_ by a PLP‐dependent enzyme, tyrosine apodecarboxylase, whose activity is directly proportional to PLP concentration. Then, tyramine oxidase catalyzed the oxidation of tyramine in hydroxybenzyl aldehyde and H_2_O_2_. Finally, H_2_O_2_ was converted in quinoneimine whose absorbance was measured at 546 nm. Intraerythrocytic PLP levels were calculated as follows:

$${\text{[PLP]}}_{\mathrm{intraerythrocytic}}=\left[{\left[\text{PLP}\right]}_{\mathrm{blood}}-\left(1-\text{Ht}{\text{[PLP]}}_{\mathrm{Plasma}}\right]/\text{Ht}\right)$$

### Statistical analysis

Collected data were described overall and by individual along time with mean ± standard deviation for continuous variables, and number, percentage for categorical data. For PK/PD studies, differences were tested using nonparametric Friedman's test. Unilateral Student's *t*‐test was performed between groups using unequal variance. Group differences in tumor growth were tested using two‐way (time and intergroup variability) ANOVA. EFS was estimated by Kaplan–Meier estimator and compared between groups log‐rank test. Level of significance was set at *P* < 0.05.

## Results

### MGL production process

**Figure 1:**
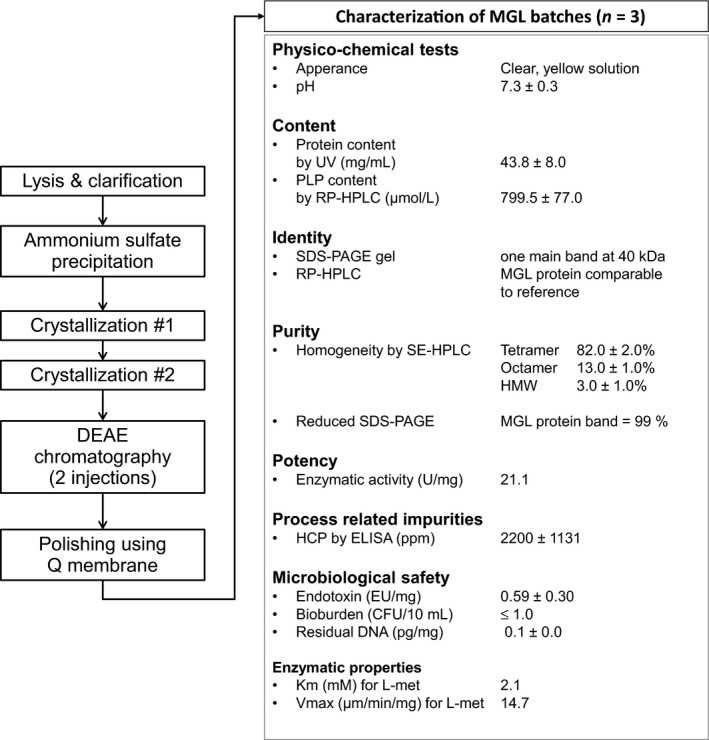
Downstream process for large‐scale purification of *Pseudomonas putida* MGL and main characteristics of the purified enzyme. Data corresponded to three representative cGMP runs. Tetrameric form is the main MGL isoform. HMW endotoxin content was evaluated by LAL method. HCP contaminants were evaluated by ELISA (Cygnus kit). *Escherichia coli* residual DNA content was evaluated by Q‐PCR. Total aerobic microbial count in (Bioburden) was carried out by membrane filtration on TSA plate and yeast plate for 5 days. Specific activity and MGL affinity for Met were determined using ammonia assay kit (Roche) as described in Materials and Methods, and *K*_m_ and *V*_max_ values were obtained by Lineweaver–Burk method. HMW, high molecular weight; HCP, host cell proteins.

The MGL transcript sequence from *P. putida* was adapted to HMS174 (DE3) host strain for large‐scale production of recombinant enzyme according to cGMP requirements. The defined method (Fig. 1) resulted in large amounts (172 ± 48 g per batch) of highly purified MGL, with a production yield of ~0.5 g/L of fermentation. The major enzyme characteristics of a representative run are reported. Enzyme release specifications were chosen in order to meet the requirements for encapsulation process and for human IV injection (sterility and low endotoxin levels). MGL was formulated in a buffer with osmolarity and pH close to physiological values and thus, fully suitable for RBC use. In proper storage conditions at ≤−70°C, the enzyme exhibited a stability of at least 12 months.

### MGL enzyme induces cytotoxicity in human cancer cell lines

**Figure 2:**
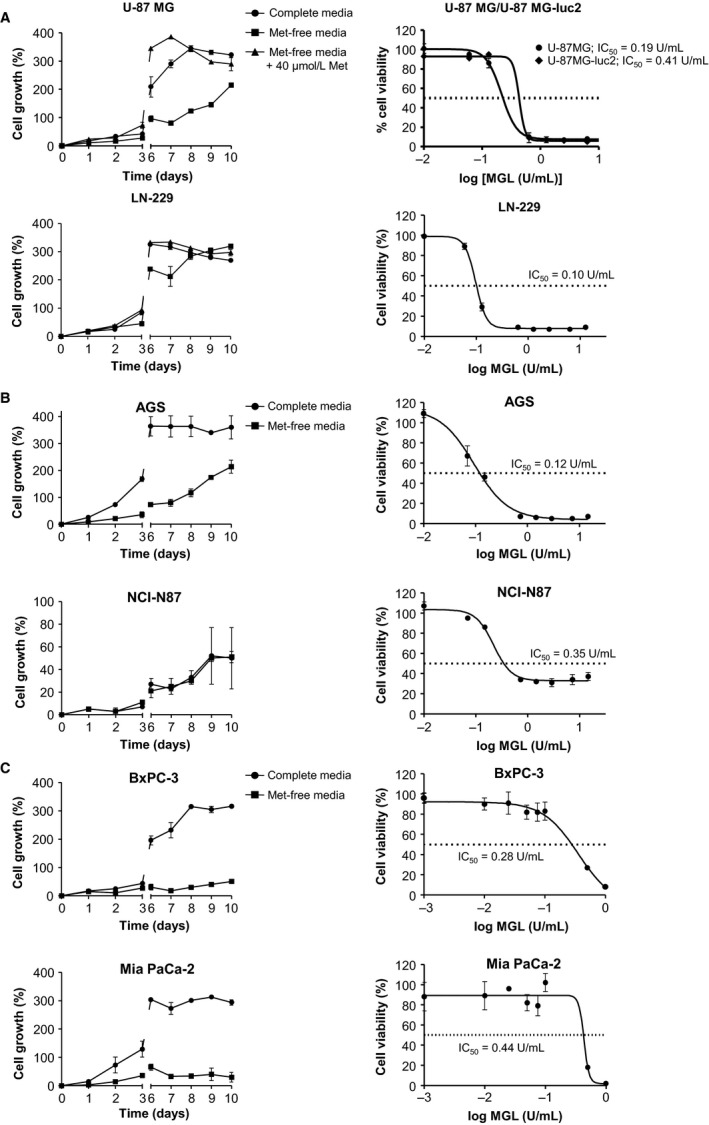
Sensitivity to Met depletion and MGL enzyme inhibition of cell viability in several cancer cell lines. Human glioblastoma (A), gastric adenocarcinoma (B), and pancreatic carcinoma (C) cell lines were placed in Met‐depleted medium and were subjected to CCK‐8 assay at different times to determine their respective viability (left panels). Effect of Met supplementation with physiological dose (40 *μ*mol/L) was also tested in glioblastoma cells. In parallel, cells lines were also treated with different concentrations of MGL for 3 or 4 days in their respective complete growth medium (right panels) and then were subjected to CCK‐8 assay. Luciferase‐tagged U‐87 MG‐luc2 glioblastoma cells (A, left graph) used for in vivo studies were also tested to verify absence of phenotype drift with the U‐87 MG parental cell line. Graphs represent data from three independent experiments. Mean ± SD.

Several arguments are in favor of a Met‐dependent phenotype of glioblastoma cells 19, 20, 32, 33. Two human glioblastoma cell lines (U‐87 MG and LN‐229) were cultured in complete and Met‐free media for 10 consecutive days (Fig. 2A, left panels). Proliferation assays showed that U‐87 MG cell growth was strongly reduced in Met‐free medium, while LN‐229 cells proliferation was not altered in this condition, despite a slight growth reduction between day 6 and day 8. For both cell lines, growth rate in Met‐free medium supplemented with a physiological concentration of Met (40 *μ*mol/L) was similar to proliferation in complete medium. All these results indicated the dependence of U‐87 MG cells on Met to grow, while LN‐229 proliferation was only slightly impaired by Met removal. The effect of purified MGL on proliferation was then investigated (Fig. 2A, right panels). MGL strongly inhibited cell growth in a concentration‐dependent manner in U‐87 MG and LN‐229 cells, with IC_50_ values of 0.19 and 0.10 U/mL, respectively. Half‐maximal inhibitory concentration was also determined in U‐87 MG‐luc2 cells and was slightly higher than for the parental cell line (0.41 U/mL).

The literature reports that gastric and pancreatic cancers harbor a Met‐dependent phenotype and are sensitive to MGL treatment 16, 22, 34, 35. Sensitivity to Met depletion and MGL cytotoxic effect were then tested on AGS and NCI‐N87 gastric carcinoma (Fig. 2B) and on BxPC‐3 and Mia PaCa‐2 pancreatic adenocarcinoma cell lines (Fig. 2C). Regarding Met sensitivity, all tested cell lines, except NCI‐N87, were dependent on Met for maintaining their proliferation rate. Gastric cancer and pancreatic adenocarcinoma cell lines were also sensitive to MGL treatment, with mean IC_50_ values of 0.24 and 0.36 U/mL, respectively.

### Erythrocytes and PN extend met depletion in vivo and improve MGL half‐life

**Figure 3:**
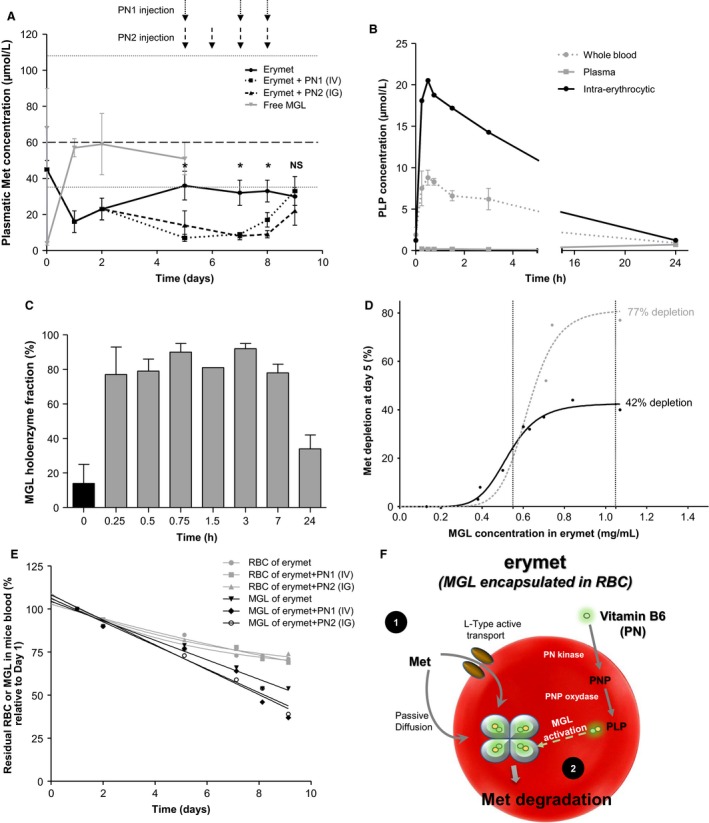
Pharmacokinetic/pharmacodynamic studies of erymet products and influence of cofactor administration in CD1 mouse model. Pharmacodynamics (A) of erymet products (11.44 U/mL) and free form MGL (11.88 U/mL) at 8 mL/kg doses in mice (*n* = 59). Met depletion by single injection of erymet alone or supplemented with PN by IV or IG route after 5 days was statistically significant (*P* < 0.05) from the mean Met concentration (based on 30 control mice) for all points except for D9 with PN1 IV injection (NS). Gray dotted lines: lowest and highest individual values observed in control mice. Conversion of PN by erythrocytes in erymet‐treated CD1 mice (B). Mice (*n* = 21) received a single injection of erymet (11.96 U/mL at 8 mL/kg) at day 0 and a single IG administration of PN (1.5 mg/mL) at day 5. PLP concentrations in whole blood, plasma, and RBC fractions (adjusted to Ht) were determined over 24 h using VB6 enzymatic assay. MGL holoenzyme proportion (C) at day 5, without PN uptake (black bar) and over 24 h after IG administration of the cofactor (gray bars). Mean ± SD. Percentage of Met depletion in mice depending on MGL concentration in erymet products (D) with (gray line) or without (black line) PN supplementation (*n* = 12). MGL concentrations were reported in mg/mL in order to compare enzyme batches with slightly different specific activities. Pharmacokinetics (E) of RBC and MGL in CD1 mice (*n* = 44) after single injection of erymet products alone or supplemented with PN. Expected mechanism of action of erymet (F): plasma Met (1) enters erythrocyte by passive diffusion and active transport. In parallel, erythrocyte enzymatic cascade converts PN provided by oral or intravenous administration into its biologically active form (PLP). Encapsulated MGL (2) degrades Met and leads to Met restriction in blood stream and tumor microenvironment.

Intravenous injection of recombinant MGL (0.9 mg/mL, equivalent to 11.88 U/mL) in CD1 mouse model (Fig. 3A) induced a rapid but transient decrease in Met plasma concentration (3.7 ± 0.6 *μ*mol/L) 15 min after administration followed by a rapid return to the physiological level (60.0 ± 17.0 *μ*mol/L; mean concentration of 30 animals from eight studies) 24 h postinjection. By comparison, erymet administration (1.05 mg/mL; 11.44 U/mL) lowered Met concentration to ∼20 *μ*mol/L at 24 h, corresponding to a 65% depletion. Met level slowly increased between day 1 and day 5, remained stable and under the lowest individual concentration observed in CD1 mice (35 *μ*mol/L) from day 5 to day 9, and corresponded to a 34% depletion of the control level after 9 days, confirming the improvement of MGL pharmacodynamics by RBC entrapment.

To determine the impact of cofactor supplementation on erymet pharmacodynamics, administration of PN precursor, by intravenous (IV) or intragastric (IG) route, was tested in vivo. PN injections were started at day 5, which corresponds to the time point when Met depletion was stabilized after erymet injection. The number of PN injections was voluntarily limited to three administrations per mouse to avoid caudal vein lesions while PN IG administrations were done daily over a 4 days period. PN concentration was extrapolated from human maximal daily dose and corresponded to 300 mg/day orally and 200 mg/day intravenously for a 70‐kg adult 36, 37. To apply the same ratio despite different number of injections, the dose of PN intravenously injected (PN1) was 2.8 mg/kg, while the PN dose for the IG route (PN2) was 3.2 mg/kg. Three hours after PN1 IV injection, Met plasma concentrations significantly decreased to 7.2 ± 1.7 *μ*mol/L, 8.5 ± 1.1 *μ*mol/L, and 17.1 ± 4.4 *μ*mol/L on day 5, day 7, and day 8, respectively (*P* ≤ 0.002). From the third PN1 IV injection (day 8), Met concentrations began to increase to reach the level observed in the erymet‐only group with 33 ± 8 *μ*mol/L on day 9, 24 h after the last PN1 injection. Three hours after PN2 IG administration, Met values also decreased rapidly to reach 14.3 ± 8.0 *μ*mol/L, 8.1 ± 2.1, and 9.0 ± 2.0 *μ*mol/L on day 5, day 7, and day 8, respectively (*P* ≤ 0.002). Even if this decrease was slower than in the erymet + PN1‐treated group on day 5, the effect on Met depletion appeared to be slightly prolonged with PN2 IG than with PN1 IV supplementation. Indeed, 24 h after the last PN injection, Met concentration of the erymet + PN2‐treated group (22.2 ± 8.3 *μ*mol/L) did not reach value of the erymet‐treated group.

To demonstrate the conversion of PN into PLP by RBC, pharmacokinetics of PLP in erymet‐treated mice following PN oral uptake was investigated over 24 h (Fig. 3B). Intraerythrocytic concentration rapidly increased and was maximal (20.5 *μ*mol/L) 30 minutes after uptake and then slowly decrease to return to initial concentration (1.2 *μ*mol/L) at 24 h. Impact of rapid intraerythrocytic conversion of PN into PLP on MGL enzyme activity was assessed in CD1 mice (Fig. 3C). At day 5, RBC contained only a low proportion (15%) of MGL holoenzyme. However, after oral PN supplementation, encapsulated MGL is found mainly (70–80%) under its active form, only 15 min after uptake. The high proportion of holoenzyme remained stable for 7 h and started to decline 24 h postadministration, confirming the accumulation of PLP in the erythrocyte.

Pharmacodynamics studies allowed precision of erymet product specifications for acceptable Met depletion (Fig. 3D). Entrapment of 0.55–1.05 mg/mL of the present MGL led to 30–77% effective depletion of mouse plasma Met concentrations 5 days after a single erymet injection associated with PN administration.

Results of pharmacokinetic studies in CD1 mice using fluorescent MGL‐containing RBC (Fig. 3E) revealed that RBC's half‐life was between 17 and 19 days, which was similar to retransfused erythrocytes survival time 38. In parallel, MGL activity in the samples was determined and revealed that the encapsulated MGL enzyme's half‐life was slightly lowered to 12 days. Surprisingly, supplementation with PN, by IV or IG route, led to a more pronounced decline of MGL enzyme survival in erymet products (8–9 days), but remained largely extended when compared to free enzyme half‐life reported elsewhere 23, 24. Erythrocytes role as natural synthesizers of PLP cofactor from PN and erymet mechanism of action on Met degradation are summarized in Figure 3F.

As erymet product pharmacokinetics seemed to be impacted by PN, we tested the influence of the cofactor on entrapped MGL and RBC half‐lives in vivo by modulating the frequency of PN administration (0, 1, and 2 daily doses), without modifying the total PN concentration administered per day (Fig. 3F). MGL half‐life decline was directly correlated to PN administration frequency, motivating the choice of 1 PN administration for animal proof of concept.

### Erymet treatment inhibits glioblastoma tumor growth in athymic nude mice

**Figure 4:**
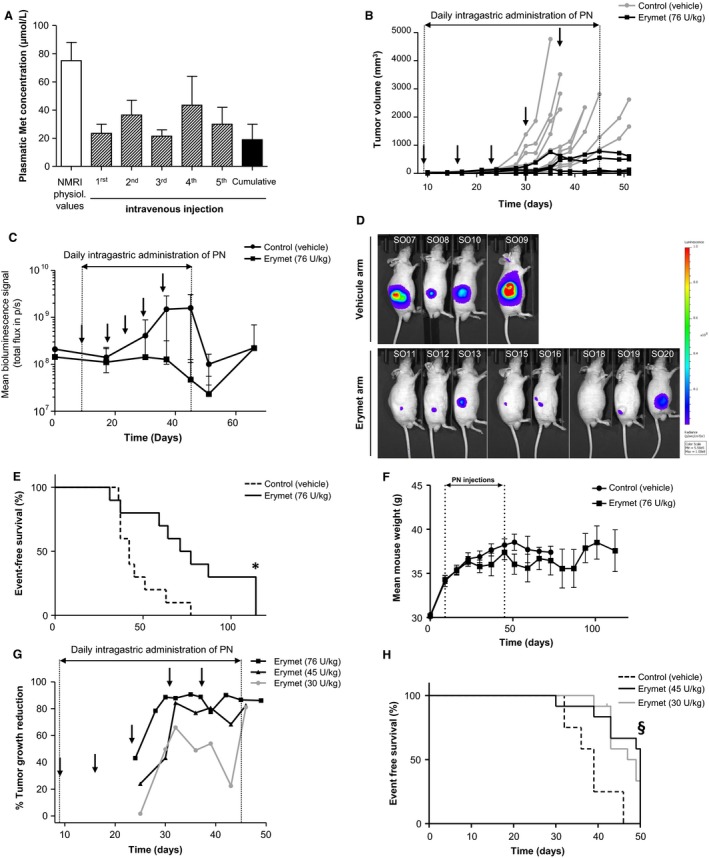
Effect of repeated intravenous administration of erymet on human glioblastoma U87‐MG Luc2 tumor xenograft growth in athymic mice. Mice were ectopically implanted (D0) with U87 MG‐Luc2 cells and, after 9 days, intravenously injected with vehicle (control) or erymet at the dose of 76 U/kg, once a week, at D9, D16, D23, D30, and D37. PN cofactor (6.7 mmol/L) was injected daily per oral route from D9 to D45. Effects of erymet on plasma methionine depletion (A), tumor volumes (B), tumor growth determined by bioluminescence imaging (C and D), and on event‐free survival (E). Data are mean of 10 mice in each group, except for L‐met concentrations (*n* = 2). Body weight measurements (F) of erymet‐ and vehicle‐treated mice. Effects of erymet treatment at reduced doses (30 and 45 U/kg) on tumor growth (G) and on EFS (H). Bars: SE. *Survival study ended at D112 and all remaining animals were sacrificed. ^§^Survival study ended at D50 and all remaining animals were sacrificed. Arrows: erymet/vehicle treatment.

Treating subcutaneous tumor‐bearing mice with a weekly single IV injection of erymet at 8 mL/kg (76 ± 17 U/kg) for 5 weeks, in association with daily PN supplementation by gavage, led to plasma Met level decrease in mice when compared to control group (75.0 ± 18.4 *μ*mol/L). Twenty‐four hours after injection, each single administration lowered Met levels to a range between 21.5 ± 6.4 and 43.5 ± 29.0 *μ*mol/L, corresponding to a 42–71% decrease in the control value. Met plasma level was decreased by 75% (19.0 ± 15.6 *μ*mol/L) after the five cumulative erymet injections (Fig. 4A). In parallel, erymet treatment during five consecutive weeks caused a marked inhibition in U‐87 MG‐luc2 tumor xenograft growth (*P* [variability] = 0.0013; *P* [time] < 0.0001) (Fig. 4B). At day 45, mean absolute tumor volume was reduced by 85% (1243.5 ± 585.5 vs. 188.7 ± 107.8 mm^3^, for vehicle and erymet, respectively). Additionally, mean bioluminescence at day 45 was reduced by 60% (12,883.9 vs. 5165.6 p/s) (Fig. 4C and D). Finally, erymet treatment of the glioblastoma tumors led to a significant (*P* = 0.0094) prolongation of EFS of the treated mice (Fig. 4E). All animals of vehicle‐treated group died or were euthanatized no later than day 77, while 30% (*n* = 3) of erymet‐treated mice were still alive at the end of the study (day 112). At this time, mice were euthanized and mean tumor volume was limited to 295.1 ± 237.5 mm^3^. Follow‐up at day 112 indicated that two animals exhibited good clinical status while the third one presented diarrhea. All along the study, the impact of erymet + PN treatment was very limited with only a slight but stabilized decrease in mice body weight in the treated group (Fig. 4F).

In addition, another in vivo study was performed to evaluate if same efficiency of erymet treatment would be observed at two lower doses (i.e., 30 and 45 U/kg) in the same mouse glioblastoma model. Significant tumor growth delay was observed from day 32 (Fig. 4G). Indeed, erymet‐treated mice at the doses of 30 ± 6 or 45 ± 9 U/kg displayed, respectively, a mean tumor volume decreased by 64% and 83% in comparison to control mice (vehicle). At day 46 (maximal evolution of tumor growth for vehicle arm), both doses display around 80% of tumor growth inhibition versus control. Survival results (Fig. 4H) and clinical observations indicated that erymet treatment (at 30 and 45 U/kg) combined with PN cofactor had a positive effect on the health of mice implanted with U‐87 MG‐Luc2 compared to vehicle. Moreover, the dose of 45 U/kg of erymet seemed to be more positive than the dose of 30 U/kg with a better survival at 50 days after tumors implantation (58% and 33% of survival, respectively), and less severe tumor necrosis development (data not shown). Significance of results was confirmed by a *P* value (log‐rank test) equal to 0.0005. In conclusion, five administrations of erymet at a dose of 45 U/kg combined to daily PN injections in subcutaneous glioblastoma‐bearing mice resulted in a significant tumor growth inhibition and improvement of lifespan over time compared to control arm (without treatment). In addition, the treatment at this dose is comparable to results obtained for the dose of 76 U/kg for the period from day 32 to day 46 holding around 80% of tumor growth reduction.

### Preliminary toxicology examination of repeated erymet injections in mice

Safety of erymet treatment was investigated in male and female CD1 mice by determining whether four IV injections at ~ 60 U/kg, at 7‐day interval, combined with daily administration of PN cofactor by oral route during 23 days induces side effects in mice. The selected dose was chosen to be well above (30% superior) the lowest efficient dose that induced tumor growth regression (i.e., 45 U/kg). Number of injections was lowered from 5 to 4 administrations in order to be in line with the treatment envisioned in clinics.

**Figure 5:**
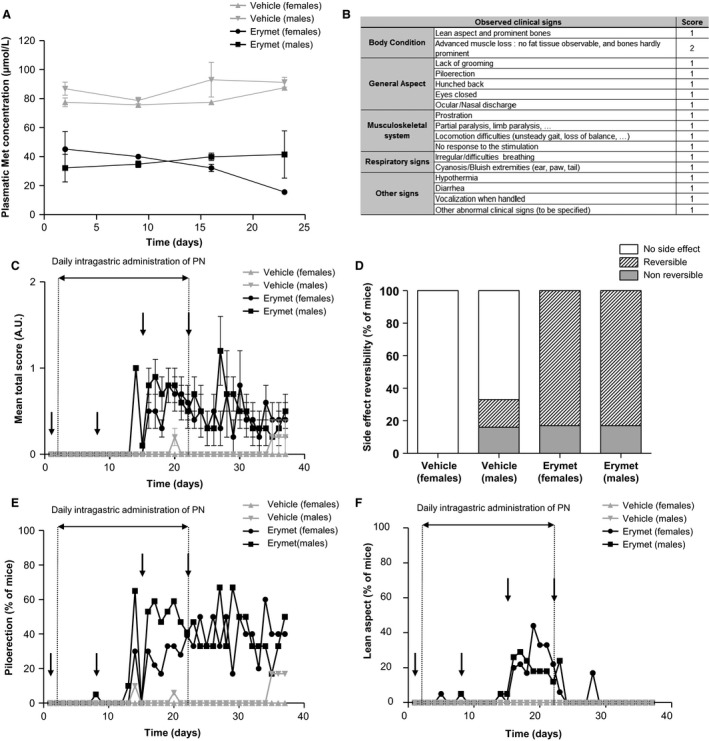
Repeated dose toxicity study of erymet combined with PN cofactor in mice. Clinical observations were recorded in erymet‐ (*n* = 32) and vehicle‐treated (*n* = 32) CD1 mice throughout both treatment (day 1 to day 22) and recovery (day 23 to day 37) periods. Plasma Met levels in mice (A) after treatment with erymet at 60 U/kg (*n* = 16) or vehicle (*n* = 16). Scoring system (B) used to establish mice clinical state and total scores (C; mean values ± SE) of all mice. Proportion of reversed side effects of treatment (D) on mice clinical state. Major clinical signs observed during the study were piloerection (E) and transient slimming (F) of the animals. Arrows: erymet/vehicle treatment. AU, arbitrary unit.

To assess the good efficacy of four IV injections of erymet coupled with daily intragastric PN administrations, plasma Met concentration was measured every following day of treatment administration (see Fig. 5A) using a dedicated group of three mice. The Met concentration measured in both males and females treated with vehicle only was globally stable from day 2 to day 23, with a mean concentration ranging from 77.5 to 87.6 *μ*mol/L in females and from 78.7 to 93.1 *μ*mol/L in males. At each time step, the l‐methionine concentration measured in treated animals was lower than for mice treated with vehicle alone, traducing a good depletion in l‐methionine induced by the treatment (~58% of mean depletion for both males and females). During the 23 days of the study and the following 15 days of recovery period, no animal mortality was observed in both vehicle and erymet groups (data not shown). A scoring grid (Fig. 5B) was used for daily clinical follow‐up of the animals and total scores measured from day 1 to day 37 were compiled in Figure 5C. During the treatment period (day 1 to day 22), 79% of mice treated with erymet + PN showed moderate side effects (i.e., a score <2) as compared to only 8% in the control group. However, during the recovery period (from day 23 to day 37; Fig. 5D), those side effects were reversed for almost all mice (83% of side effects reversal was observed for both males and females). Proportion of nonreversible side effects at the end of the recovery period was similar in both treated groups (17% for males and females), while vehicle‐treated group harbored 0% (no side effect) and 16% for females and males, respectively.

The major clinical signs observed throughout the study were piloerection (in 40–50% of the animals; Fig. 5E) and a transient lean aspect of the animals (Fig. 5F). Minor clinical signs in treated animals were hunched back, prostration, low response to the stimulation, and lack of grooming, but were not considered as significant due to their short and transient observation. Hematological and serum analysis indicated a slight decrease in hemoglobin and triglyceride concentrations maintained during the 14 days of recovery period after treatment ending, and a transient increase in globulin concentration (data not shown). When pooled together, all these results clearly revealed that erymet treatment at 60 U/kg 4 times at 7 days interval and combined with daily administration of PN cofactor harbored a good safety profile in mice without inducing severe side effects.

## Discussion

l‐Methionine dependency is now considered the only known general metabolic defect in cancer 13. Despite the confirmed efficacy in several preclinical tumor‐bearing models, phase I clinical studies highlighted the difficulties of setting up and maintaining a Met‐free diet for long‐term depletion in cancer patients without deleterious effects 17, 18, 19, 39. Intravenous injection of Met‐catabolizing enzymes as MGL provides an efficient alternative to alimentary Met restriction. Based on this existing rational, a MGL manufacturing process was developed to obtain a highly purified enzyme at an industrial scale that meets the cGMP requirements and that is fully compatible with human injection. A downstream purification method was designed to obtain lower levels of contaminants (host cell proteins, residual DNA, endotoxins) and to our knowledge, this is the first time a highly purified MGL production process fully compatible with phase I clinical trial prerequisites has been described.

The major limitations associated with MGL use are a short half‐life in the bloodstream and immunological reactions 40, 41. The current study clearly demonstrated that encapsulation of MGL in RBC can overcome these problems and offers a suitable solution for therapeutic use, due to the intrinsic properties of erythrocytes. Met enters erythrocytes by passive diffusion (main method) and neutral amino acids L‐type transporter, leading to a Met concentration in RBC (∼30 *μ*mol/L) that is similar to the Met concentration in plasma 42, 43, which allows continuous substrate delivery to entrapped MGL enzyme. Moreover, encapsulated MGL's activity is highly dependent on the presence of its cofactor. Hence, the PLP's bioavailability is a key regulator of erymet function. In addition, to protect MGL from degradation and from recognition by the immune system, erythrocytes actively participate in MGL function as they provide the whole machinery to synthesize PLP from PN 27. Indeed, RBC are known as a natural pool of PLP, which therefore supports the interest in using them as an MGL carrier. More generally, we can assume from this characteristic that erythrocyte is the only system reported to date that is compatible with effective administration of PLP‐dependent enzymes. Pharmacokinetic and pharmacodynamic results presented herein clearly demonstrated the direct implication of RBC in MGL half‐life increase (from a few hours to 8–9 days) and in rapid conversion of PN uptake to biologically active PLP, which led to effective and prolonged plasma Met depletion in vivo. Another interesting property of erymet resulting from the use of erythrocyte enzyme cascade is the possibility of adjusting plasma Met levels by modulating PN intake. As MGL absolutely requires a concomitant oral vitamin B6 supplement for its catabolizing activity, erymet thus offers a controlled mechanism that avoids any adverse patient reactions, ensuring this therapeutic approach's safety.

Another advantage of the enzyme‐loaded erythrocytes approach is avoiding hypersensitive reactions in patients by shielding the bacterial enzyme from the immune system. The tolerability, enzyme activity prolongation, and clinical benefits of RBC as enzyme carriers were clearly demonstrated in a recent phase III clinical trial on primary relapse/refractory acute lymphoblastic leukemia 44.

As Met is an essential amino acid involved in several metabolic pathways, including protein synthesis, protection from oxidative stress, nuclear and cell division, and DNA methylation 15, we hypothesize that a total Met depletion at the systemic level could be harmful for patients and that a controlled partial depletion sustained for several days or weeks is preferable to induce cancer cell death without damaging normal cells.

Among Met‐dependent tumors, glioblastoma (GBM) was identified as a potential candidate for erymet therapy, as several clinical arguments support the interest in using Met deprivation therapy in patients suffering from these highly aggressive primary brain tumors. Indeed, radiolabeled Met (MET‐PET) uptake is one of the metabolic imaging tools used to determine brain tumor volumes and to identify residual tumors after resection or recurrent gliomas 10. Studies also demonstrated that Met uptake is highly increased in viable glioma tumors cells, but remains low in macrophages and other normal cells 45. In the same way, a study investigating the correlation between Met uptake and glioma features in 194 patients concluded that malignant pathological features were detected in the areas with the highest Met uptake 46. Another argument in favor of GBM targeting by erymet therapy is the hypervascularization state of this tumor type: in gliomas, the spectrum of low‐ to high‐grade tumors reflects major differences in vasculature as the low‐grade to high‐grade transition is characterized by neovascularization, an adaptive phenomenon known as the “angiogenic switch.” This event allows grade II gliomas, characterized by a linear growth phenotype and the absence of microvascular proliferation, to evolve to glioblastoma, which is defined by exponential growth and rapid clinical decline following high rate microvascular proliferation 47, 48. Current in vitro studies on human GBM cell lines reported that U‐87 MG cells (parental or luciferase tagged) were auxotroph for Met. Using a subcutaneous xenograft mouse model of GBM, we were able to demonstrate that five IV injections (76 U/kg) of MGL‐loaded erythrocytes induced a sustained depletion of plasma Met (between 40% and 70% of control after each injection) and displayed a substantial antitumor activity. We also demonstrated that repeated injections reduced doses of erymet (40 and 30 U/kg) induced the same tumor growth inhibition in that GBM mouse model.

In parallel, a preclinical toxicity study in CD1 mice underlined the safe profile of repeated injections of erymet combined with PN administration. Pooled together, these preclinical results on erymet efficacy and safety will help us to anticipate extrapolation of efficient doses in clinics.

MGL from *P. putida* was reported to possess the ability to partially catabolize l‐homocysteine or l‐cysteine 49, 50. We can assume from these studies that the strong cytotoxic effect of MGL observed on the practically Met‐independent LN‐229 cell line was due to its affinity for one secondary substrate. Further investigations are ongoing to determine the full spectrum of our MGL enzyme's specificity.

In conclusion, we reported here that erymet, a RBC‐encapsulated form of MGL, acted as an intravascular microbioreactor, and effectively led to a long‐term, sustained significant plasma Met depletion in vivo, resulting in an efficient tumor reduction growth in a GBM tumor‐bearing mouse model.

By limiting the intracellular level of Met, erymet is expected to alter several cellular pathways. Several works recently reported that dietary or enzymatic Met depletion caused a cell cycle arrest, preponderantly in G_2_/M phase, and forced cell to undergo apoptosis 13. Investigations on erymet action mechanism on cancer cells are planned and will help to determine the possible impact on cell cycle arrest, DNA methylation, and the effect of erymet on transsulfuration pathway and oxidative stress.

Erymet product manufacturing is currently being scaled up for human trial purposes. The osmotic‐based method using ERYcaps^®^ technology offers a robust and reproducible process for entrapping therapeutic enzymes in human erythrocytes and has already been proven to provide clinical therapeutic benefits in the case of L‐asparaginase, as compared to the free enzyme.

Regulatory toxicity studies (Good Laboratory Practice compliant) is planned for end of 2017 and if the safety profile on animals is confirmed, a phase I clinical trial will be set up to demonstrate the optimal tolerability and biological efficacy of erymet associated with PN administration in patients with cancer potentially sensitive to Met deprivation.

## Conflict of Interest

None declared.
